# Supplementary material for: Elucidating design principles for Ribozyme-Enabled Tissue Specificity (RETS) to allow precise expression without specialized promoters
Source: bioRxiv. 2025 Sep 22:2025.08.14.670357. Originally published 2025 Aug 14. Preprint. [Version 2] doi: 10.1101/2025.08.14.670357 (PMC12363958; doi:10.1101/2025.08.14.670357)
Supplement: 1 [file NIHPP2025.08.14.670357V2-supplement-1.pdf]

bioRxiv preprint doi: https://doi.org/10.1101/2025.08.14.670357; this version posted September 22, 2025. The copyright holder for this preprint (which was not certified by peer review) is the author/funder, who has granted bioRxiv a license to display the preprint in perpetuity. It is made available under aCC-BY 4.0 International license.

Supplementary Materials

Supplemental RETS construct design

For figure 2, RETS constructs were designed to detect the coding sequence of an *Arabidopsis*-codon-optimized Cas9 transcript. Guide RNA sequences were 200 bases in length, and the mScarlet cargo transcript was split at the start codon. Upstream of the start codon was either the complete 67-base omega enhancer (RETS_Omega) or just the final 4 bases of the enhancer to provide a complete IGSBS (RETS_NO-Omega). A third, target construct was prepared in parallel which expressed Cas9 under control of the *Arabidopsis* UBQ10 promoter, terminated by tHSP.

For figure 3, the RETS construct was re-designed to target exon 1 of *Arabidopsis* Sucrose transporter 1, AtSUC1. For figure 4, to reduce the likelihood of inducing dsRNA-dependent RNA silencing, RETS constructs with shorter guides were produced. Targeting the same region of AtSuc1, 3 constructs were made that utilize pairs of 30nt, 25nt, and 20nt RNA guide arms instead of the 200nt guide arms of the initial construct. In addition, 3 more constructs were designed that had 30, 25, and 20nt guide arms re-targeted to a region of AtSuc1 that is more divergent from the other members of the Suc gene family (see figure S2).

To facilitate more rapid prototyping, a constitutive copy of the AtSuc1 cDNA was cloned into a binary vector under control of the CaMV 35s promoter. This construct allowed for characterization of the new Suc1:RETS constructs in *N. benthamiana* leaves with transient expression via agroinfiltration.

For the PAP1 and SAP-detecting constructs in figure 5, the backbone of the cassettes was modified slightly: The guides were all made to be 50nt in length, and the split-site of the cargo was designed to be U197 of mScarlet, the second base of F66. This change altered the IGSBS, and thus the IGS of the TtRz was modified to be 5'-GACUGC-3' to reflect that change, allowing for successful reconstitution of the TtRz P1 helix, facilitating the splicing reaction.

For figure 6, Autoactive DELLA protein mutant GAI(*rht*) was constructed by amplifying *Arabidopsis* GAI1 (AT1G14920) from *Col-0* without the first 55 amino acids (using codon for M56 as the new start), analogous to what was described by Willige et al (2007). The RETS construct designed to detect KNAT2 was redesigned to reconstitute GAI(*rht*), splitting the transcript at U167, and having an IGSBS and IGS of 5'-GAUUCU-3' and 5'-GGAAUC-3' respectively. The constitutive Venus cassette was included to facilitate selection-free identification of transformants.

bioRxiv preprint doi: https://doi.org/10.1101/2025.08.14.670357; this version posted September 22, 2025. The copyright holder for this preprint (which was not certified by peer review) is the author/funder, who has granted bioRxiv a license to display the preprint in perpetuity. It is made available under aCC-BY 4.0 International license.

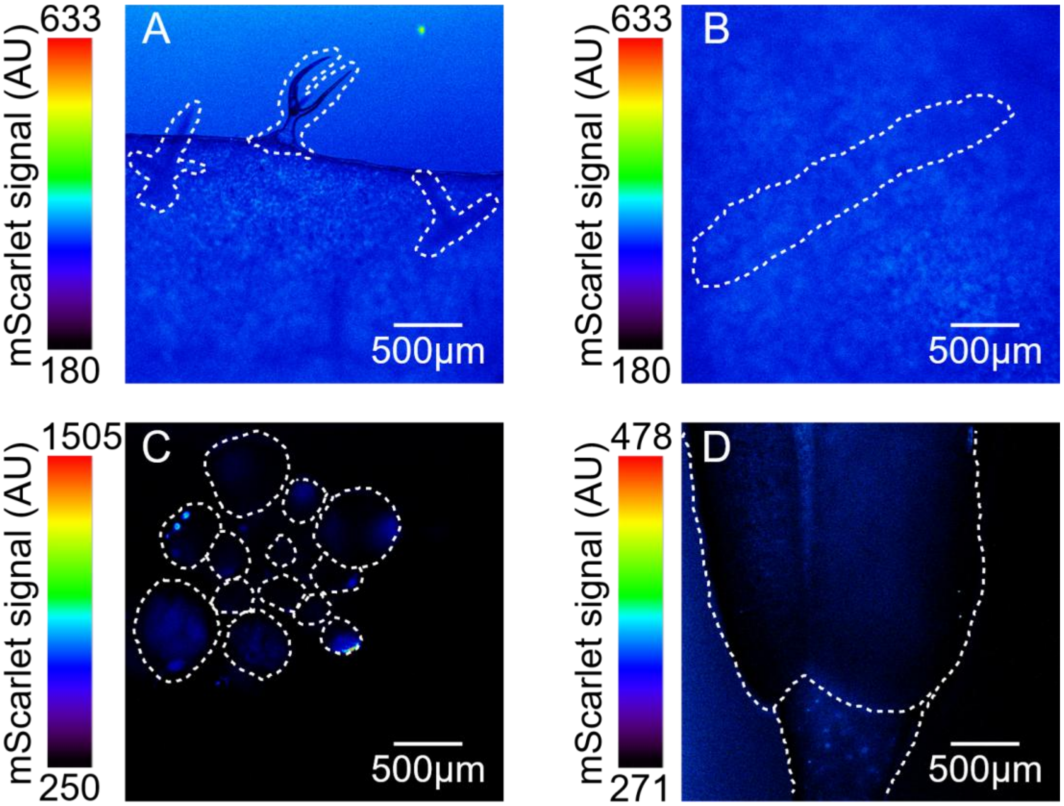

Supplemental Figure S1. Representative *A. thaliana* Col-0 wildtype fluorescent microscope images using the mScarlet channel. A) Shows wildtype trichomes, compare to Figure 3B-D, 3F and 4C. B) wildtype vasculature, compare to Figure 4D. C) Shows developing wildtype flower buds, compare to Figure 5B. D) Shows wildtype adult flower sepals, compare to Figure 5D. Displayed lookup table thresholds are calibrated to the respective experimental images they are compared to.

bioRxiv preprint doi: https://doi.org/10.1101/2025.08.14.670357; this version posted September 22, 2025. The copyright holder for this preprint (which was not certified by peer review) is the author/funder, who has granted bioRxiv a license to display the preprint in perpetuity. It is made available under aCC-BY 4.0 International license.

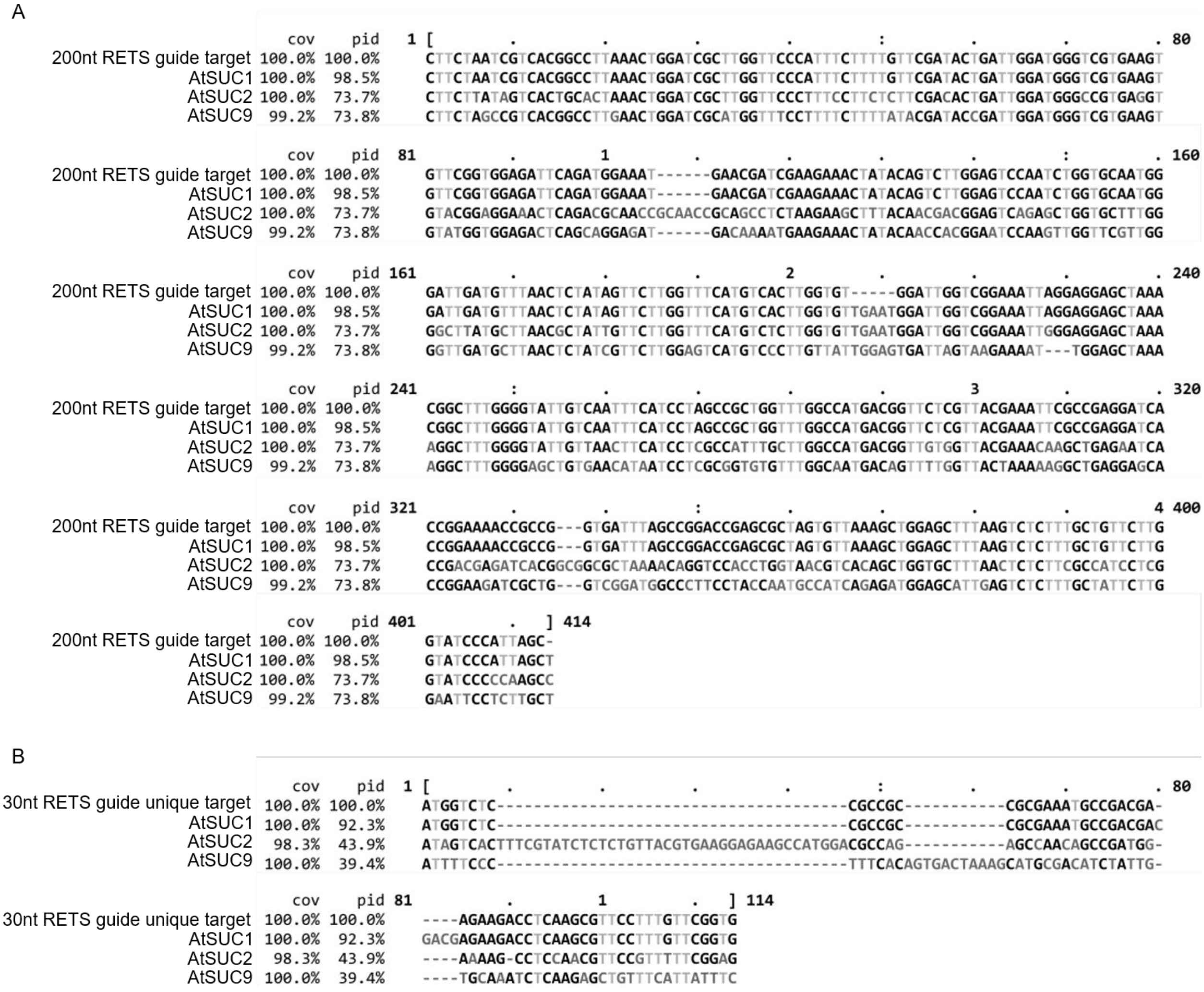

Supplemental Figure S2. Multiple sequence alignment of RETS target sequences and SUC family genes. A) Multiple sequence alignment of the original RETS construct target sequence with 200-base guides targeting a region of AtSUC1. Target sequence is aligned to the homologous region of AtSUC1 and the major vascularly-localized members of the SUC family, SUC2 and SUC9. B) Alignment of a re-designed target site for a RETS construct with 30-base guides and SUC1, 2, and 9. Note that in both alignments, there is an intentional 5-base gap in the intended target (SUC1) not covered by the RETS guides, this allows for steric space between the two 200 or 30-base guide arms. Alignments were done using Clustal Omega and visualized with MView.

bioRxiv preprint doi: https://doi.org/10.1101/2025.08.14.670357; this version posted September 22, 2025. The copyright holder for this preprint (which was not certified by peer review) is the author/funder, who has granted bioRxiv a license to display the preprint in perpetuity. It is made available under aCC-BY 4.0 International license.

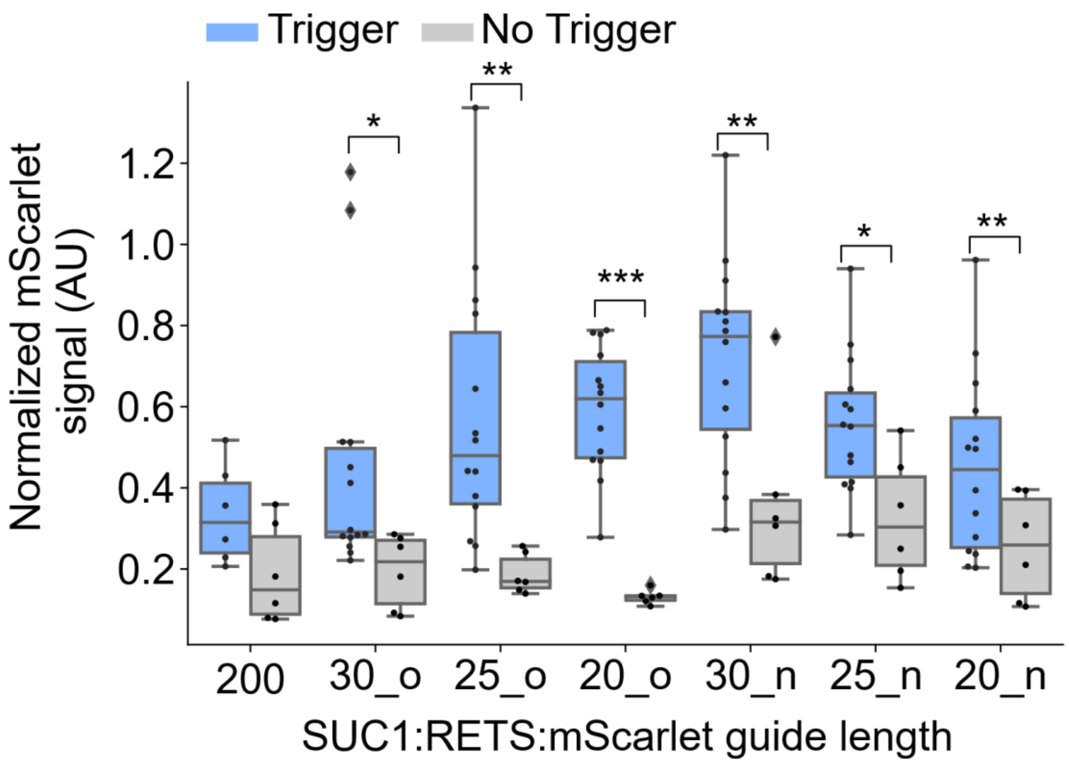

Supplemental Figure S3. Transient expression analysis of different sized RETS guides targeting AtSUC1. Experiments were done by co-agroinfiltrating the various constructs into *Nicotiana benthamiana* leaves with or without an exogenous copy of p35S:AtSUC1 (Trigger). Blue boxplots represent data from leaf discs co-infiltrated with the trigger plasmid, grey boxplots represent leaf discs that did not have the trigger co-infiltrated with the RETS construct. Y-axis represents the normalized mScarlet fluorescence signal, i.e. the SUC1:RETS:mScarlet construct mScarlet fluorescence divided by the constitutive Venus fluorescence that was measured simultaneously. Each point represents the data from an individual leaf disc. The letter after the underscore on the x-axis label represents whether the guide sequence targets the old site (o) -the same site as the 200-base construct- or the new site (n), the site that more specifically binds to SUC1 with less cross-reactivity to SUC2/9. Statistical significance determined by Students' t-test: * = p ≤ 0.05; ** = p ≤ 0.005; *** = p ≤ 0.0005.

bioRxiv preprint doi: https://doi.org/10.1101/2025.08.14.670357; this version posted September 22, 2025. The copyright holder for this preprint (which was not certified by peer review) is the author/funder, who has granted bioRxiv a license to display the preprint in perpetuity. It is made available under aCC-BY 4.0 International license.

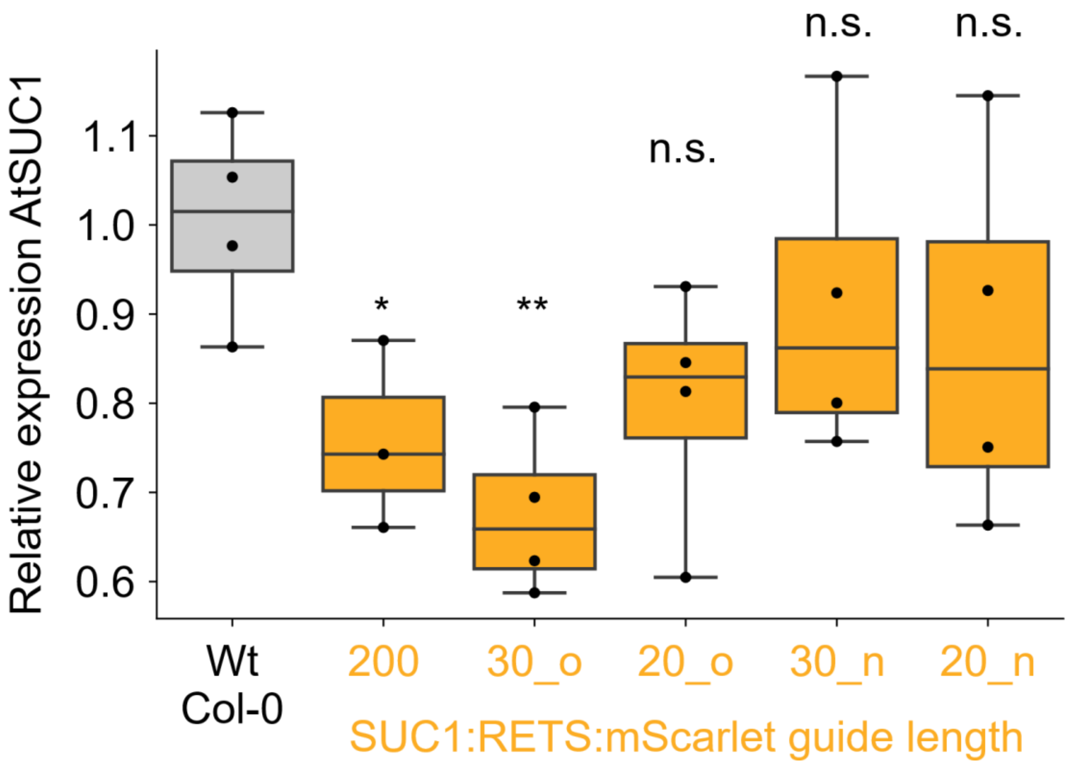

Supplemental Figure S4. Quantitative RT-PCR measurements of AtSUC1 for seedlings of Col-0 and SUC1:RETS:mScarlet lines with different guide lengths. The grey boxplot represents the distribution of SUC1 expression in wildtype Col-0 seedlings, the orange boxplots represent the SUC1 expression from SUC1:RETS:mScarlet lines. Each point represents a biological replicate of 5-8 pooled seedlings. The letter after the underscore on the x-axis label represents whether the guide sequence targets the old site (o) -the same site as the 200-base construct- or the new site (n), the site that more specifically binds to SUC1 with less cross-reactivity to SUC2/9. Statistically significant differences to Col-0 determined by Students' t-test: * = p ≤ 0.05; ** = p ≤ 0.005; *** = p ≤ 0.0005.

bioRxiv preprint doi: https://doi.org/10.1101/2025.08.14.670357; this version posted September 22, 2025. The copyright holder for this preprint (which was not certified by peer review) is the author/funder, who has granted bioRxiv a license to display the preprint in perpetuity. It is made available under aCC-BY 4.0 International license.

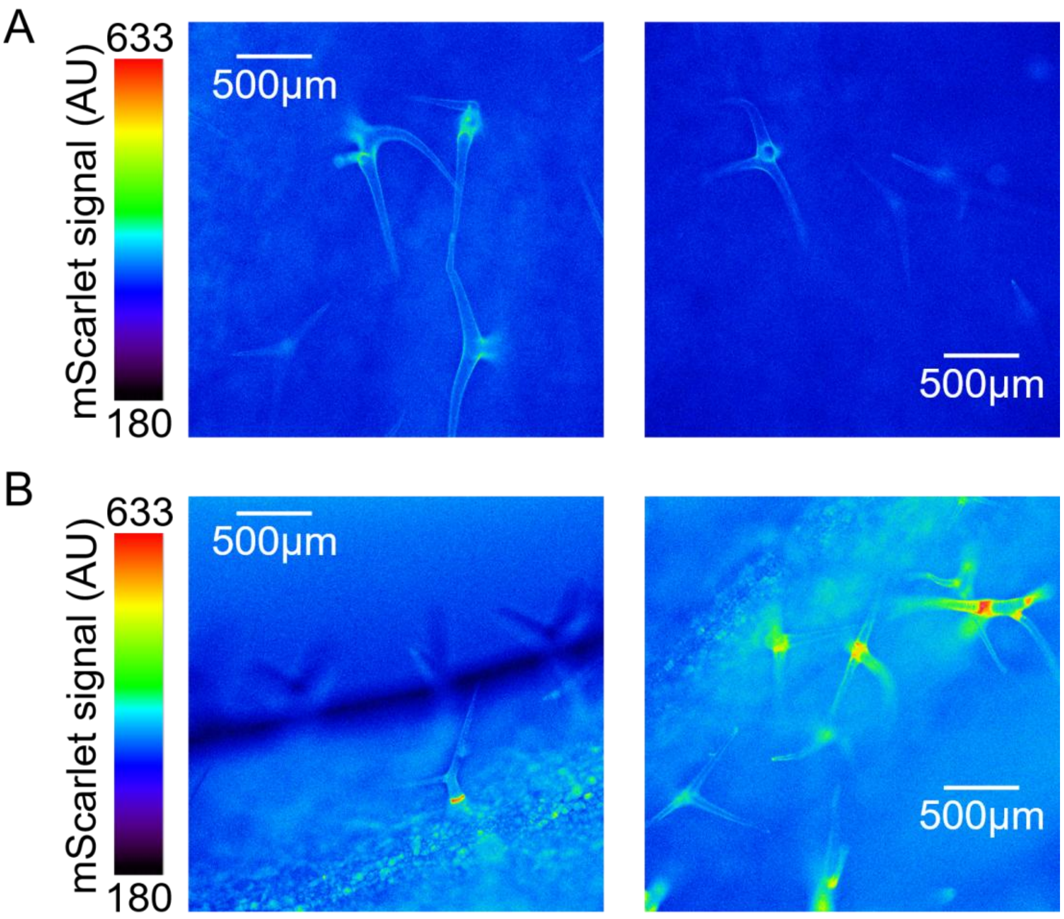

Supplemental Figure S5. Additional microscope images of SUC1:RETS:mScarlet lines with shorter guides directed to a more unique site in AtSUC1. Images were taken of adult rosette leaves with the mScarlet channel, focusing on the trichomes. A) Images of a line with 25-base guides. B) Images of a line with 20-base guides.

bioRxiv preprint doi: https://doi.org/10.1101/2025.08.14.670357; this version posted September 22, 2025. The copyright holder for this preprint (which was not certified by peer review) is the author/funder, who has granted bioRxiv a license to display the preprint in perpetuity. It is made available under aCC-BY 4.0 International license.

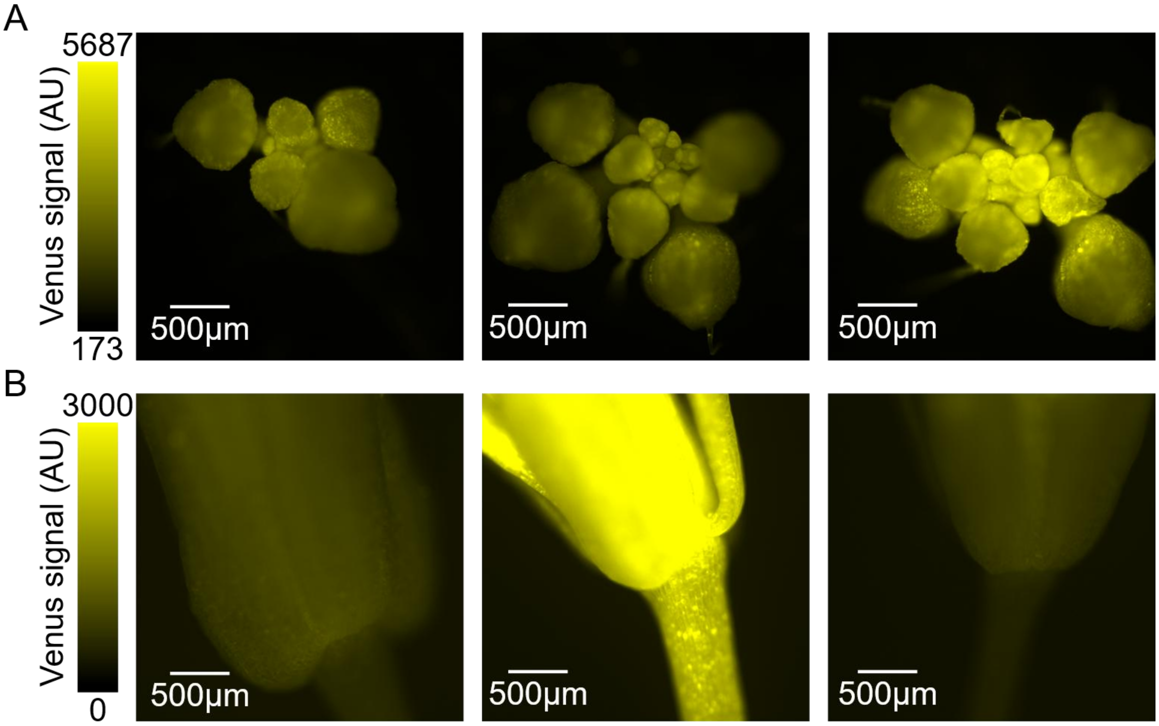

Supplemental Figure S6. Images of SAP and PAP1:RETS:mScarlet lines in the Venus channel to show uniform distribution of the T-DNA containing the RETS cassettes. A) Developing flower buds of 3 independent SAP:RETS:mScarlet lines with constitutive Venus expression. Compare to the tissue-specific distribution of mScarlet in Figure 5B. B) Adult flower sepals of 3 independent PAP1:RETS:mScarlet lines with constitutive Venus expression. Compare to the tissue-specific distribution of mScarlet in Figure 5D.

bioRxiv preprint doi: https://doi.org/10.1101/2025.08.14.670357; this version posted September 22, 2025. The copyright holder for this preprint (which was not certified by peer review) is the author/funder, who has granted bioRxiv a license to display the preprint in perpetuity. It is made available under aCC-BY 4.0 International license.

Supplemental Table S1. Plasmid constructs used in this work.

Plasmid code (P#)	Plasmid name	Plasmid use	Plasmid map link
P354	pTRANS_230d-pUBQ10:NLS-AtCas9:tHSP - pGmUbi:NLS-MCP-DREB2A-tUBQ1 - p35s:NLS-PCP-TPLN300:tNos	Cas9 Expression	https://benchling.com/s/seq-G4OTRmGF2rlvtg1XD3n8?m=slm-YpMR0Gk0KZGiCgmfjprl
P1699	Cas9_RETS-fixedIGS/IGSBS_Omega	Cas9:RETS:mScarlet with Omega enhancer	https://benchling.com/s/seq-yFpTyqR8Lbqq0ENl2P0L?m=slm-a1ATqcjVP8cdO6y94aKL
P1700	Cas9_RETS-fixedIGS/IGSBS_NO-Omega	Cas9:RETS:mScarlet NO Omega enhancer	https://benchling.com/s/seq-EjioHrHC792M8dNK78ps?m=slm-rBe20gX1IW9HGKFsIuKX
P1574	TtRz-AtSuc1-sensing(FIXED-IGS_and_IGSBS)_mScarlet-output_Venus-norm	SUC1:RETS:mScarlet<br>200nt guides	https://benchling.com/s/seq-hRIp6xrNZB73xSatXqlY?m=slm-XLVQzwZY1GL198ZUiWrr
P1735	AtSuc1-RETS_mScarlet-output_30baseGuides_oldTarg	SUC1:RETS:mScarlet<br>30nt guides	https://benchling.com/s/seq-gmR1CuYqzcwmf75gDk5E?m=slm-QP5bxh5DTAYFraO1FKHN
P1736	AtSuc1-RETS_mScarlet-output_25baseGuides_oldTarg	SUC1:RETS:mScarlet<br>25nt guides	https://benchling.com/s/seq-pYdtpCcHJk0hnL8tiEyV?m=slm-DGTS8KwQcXewqRwwTs7U
P1737	AtSuc1-RETS_mScarlet-output_20baseGuides_oldTarg	SUC1:RETS:mScarlet<br>20nt guides	https://benchling.com/s/seq-LzkQTRKXtgF89wDO0L3G?m=slm-vLlTlLNva4I2QbFxbSv5
P1738	AtSuc1-RETS_mScarlet-output_30baseGuides_NEWTarg	SUC1:RETS:mScarlet<br>30nt guides NEW TARGET	https://benchling.com/s/seq-a7OpThgtvFLvTbqXsvBs?m=slm-1KtrrGL8oP34IAqiRBi3
P1739	AtSuc1-RETS_mScarlet-output_25baseGuides_NEWTarg	SUC1:RETS:mScarlet<br>25nt guides NEW TARGET	https://benchling.com/s/seq-1BeQ0mDyoOWyN3EJvnvE?m=slm-jY3uwxDvKNdPgGFiFluZ
P1740	AtSuc1-RETS_mScarlet-output_20baseGuides_NEWTarg	SUC1:RETS:mScarlet<br>20nt guides NEW TARGET	https://benchling.com/s/seq-8ayahgGo17DGo9LFdbRK?m=slm-6QFkbTVMqcGZVc0ZSfwH
P1800	p35s_AtSuc1(No-Introns)_tHSP	Exogenous trigger for SUC1:RETS:mScarlet agroinfiltration	https://benchling.com/s/seq-Q37qKbA9Vghg6uels8pm?m=slm-iRgBCT0Gz0DlP91046v1
P1696	AtPAP1-RETS_Split-mScarlet-output	PAP1:RETS:mScarlet<br>50nt guides	https://benchling.com/s/seq-pXjzRAe2eMAFp13touu8?m=slm-9Vn6hEtIVxOs4JflzNNu

bioRxiv preprint doi: https://doi.org/10.1101/2025.08.14.670357; this version posted September 22, 2025. The copyright holder for this preprint (which was not certified by peer review) is the author/funder, who has granted bioRxiv a license to display the preprint in perpetuity. It is made available under aCC-BY 4.0 International license.

P1697	AtSterileApetala-RETS_Split-mScarlet-output	SAP:RETS:mScarlet 50nt guides	https://benchling.com/s/seq-XgQMqqNkkeOoKySGQAlu?m=slm-g2Aenj7zfU4nSxCsK3Xe
P1781	Knat2-targeting_RETS-GAIrht-output	KNAT2:RETS:GAIrht 50nt guides	https://benchling.com/s/seq-b6doB1c35mnhimuNpxWr?m=slm-YbSZo7q1F5DN5tXN5mUH

bioRxiv preprint doi: https://doi.org/10.1101/2025.08.14.670357; this version posted September 22, 2025. The copyright holder for this preprint (which was not certified by peer review) is the author/funder, who has granted bioRxiv a license to display the preprint in perpetuity. It is made available under aCC-BY 4.0 International license.

Supplemental Table S2. Primers used in this work.

Primer code (O#)	Primer name	Primer use	Primer sequence
O2058	qPCR-AtSuc1(exon1)-f1	AtSUC1 qPCR	TCCCATGTGGATGCTTCTAATC
O2059	qPCR-AtSuc1(exon1)-r1	AtSUC1 qPCR	CACGACCCATCCAATCAGTATC
o2800	qPCR_PAP1-f	AtPAP1 qPCR	CCTACAACACCGGCACTAAA
o2801	qPCR_PAP1-r	AtPAP1 qPCR, gene specific cDNA synthesis	TCCAAGGCATGGAGGATTAAC
o843	TMV-5UTR_mScarlet-qPCR-f	mScarlet qPCR	CCAACAACAACAAACAACAAACAAC
o844	TMV-5UTR_mScarlet-qPCR-r	mScarlet qPCR	GATCCTTCCATGTGGACCTTAAA
o273	[PP2A]_q-f	Arabidopsis qPCR housekeeping gene, PP2aa3 (At1G13320)	AACGTGGCCAAAATGATGC
o274	[PP2A]_q-r	Arabidopsis qPCR housekeeping gene, PP2aa3 (At1G13320)	AACCGCTTGGTCGACTATCG
o275	[PP2A]-r	PP2aa3 gene specific cDNA synthesis	CTAGACATCATCACATTGTCAATAG
o2805	qPCR_mScarlet-acrossF66split-r2	mScarlet gene specific cDNA synthesis (across F66 RETS split)	TTATAAAGGCACGACTGCCATAC
o2318	BsaI-CATG_AtGAI(dFirst55aa)-f	Amplifying the front section of GAIrht from Col-0	aaggtctcaCATGATGTCTAATGTTCAAGAAGACGATCTTTC
o2319	AtGAI(dFirst55aa)_BsaI-TTCT-r	Amplifying the front section of GAIrht from Col-0	aaggtctcaAGAATCGCGTCACCGG
o2323	BsaI-CTCG_BackHalf-GAI(rht)-f	Amplifying the back section of GAIrht from Col-0	aaggtctcaCTCGCAATCAGTTCGCTATCGATTCGG
o2324	BackHalf-GAI(rht)_BsaI-ATAT-r	Amplifying the back section of GAIrht from Col-0	aaggtctcaATATCTAATTGGTGGAGAGTTTCCAAGC

bioRxiv preprint doi: https://doi.org/10.1101/2025.08.14.670357; this version posted September 22, 2025. The copyright holder for this preprint (which was not certified by peer review) is the author/funder, who has granted bioRxiv a license to display the preprint in perpetuity. It is made available under aCC-BY 4.0 International license.

Supplemental Table S3. Arabidopsis target genes used in this work.

Gene name	TAIR accession #
SUC1	AT1G71880
SUC2	AT1G22710
SUC9	AT5G06170
SAP	AT5G35770
PAP1	AT1G56650
KNAT2	AT1G70510
GAI1	AT1G14920
